# The Protein Subcellular Mislocalization in Human Cancers

**Published:** 2020

**Authors:** Fatemeh Ghaemimanesh

**Affiliations:** Monoclonal Antibody Research Center, Avicenna Research Institute, ACECR, Tehran, Iran

Several sets of proteins exist in eukaryotic cells wherein accurately localized in appropriate cellular locations containing plasma membrane, cytoplasm, nucleus and different membrane-enclosed organelles. Each protein is involved in distinct biochemical processes within the normal cell; therefore, the correct subcellular localization of protein is vital; as it provides the physiological context for protein function. However, protein mislocalization described as changing the appropriate subcellular localization of protein, was reported as a key feature of many proteins in a variety of human cancers 1. Mislocalization has important implications in alteration of activation condition, biological function and interaction network of a protein. Particularly, the aberrant localization of tumor-suppressor proteins and proto-oncoproteins can alter their functions, respectively in either suppressing or supporting the cancer initiation in normal cells whereby cancer development, metastasis and drug resistance are increased 2.

The mechanisms by which subcellular mislocalization is arising in cancer are various and deeply reviewed by Wang and Li 2. They can be described as modification of signals directing proteins into a particular location, dysregulation of sorter and transporter machinery, Endoplasmic Reticulum (ER) retention of misfolded proteins, aberrant endocytosis and vesicular trafficking, dysregulation of signal transduction and protein post-translational modification and so on 2.

The aberrant subcellular position of proteins in cancer tissues has been investigated broadly by antibody-based strategies including Immunohistochemistry (IHC). The major localization of sortilin on cell surface of ovarian carcinoma tissues rather than the main resident in ER-Golgi compartment of normal tissues was detected using IHC technique 3. The new method of Dissociable Antibody Microarray (DAMA) combining the power of IHC staining with protein microarray, provides the facility of high-throughput detection of protein subcellular localization 4.

The property of malignant cells in differently subcellular localization of proteins might be recruited as an intelligent strategy for detection of malignant but not normal cells in clinical diagnostic and prognostic applications 5. For instance, fibromodulin, a type of proteoglycan resides in extracellular matrix, has been irregularly located on cell surface of Chronic Lymphocytic Leukemia (CLL) cells but not in normal samples by which a new CLL diagnostic biomarker appropriate for cell surface flow cytometric detection might be suggested 6. Moreover, directly targeting of locations in where proteins are accumulated in cancer cells plus selecting the structural dissimilarities of proteins by biologic weapons help us to specifically eradicate malignant but not normal cells.

In conclusion, the subscellular mislocalization of proteins is a frequent event in cancers defined as an accessory approach for proliferation, survival and invasion of tumors. However, targeting and trapping proteins in specific cellular compartments has been conceptualized as a promising approach for diagnostic, prognostic and therapeutic clinical use.
